# Carbon Dioxide Flux from Rice Paddy Soils in Central China: Effects of Intermittent Flooding and Draining Cycles

**DOI:** 10.1371/journal.pone.0056562

**Published:** 2013-02-20

**Authors:** Yi Liu, Kai-yuan Wan, Yong Tao, Zhi-guo Li, Guo-shi Zhang, Shuang-lai Li, Fang Chen

**Affiliations:** 1 Laboratory of Aquatic Botany and Watershed Ecology, Wuhan Botanical Garden, Chinese Academy of Sciences China, Wuhan, China; 2 Institute of Plant Protection and Soil Fertilizer, Hubei Academy of Agricultural Sciences, Wuhan, China; University College London, United Kingdom

## Abstract

A field experiment was conducted to (i) examine the diurnal and seasonal soil carbon dioxide (CO_2_) fluxes pattern in rice paddy fields in central China and (ii) assess the role of floodwater in controlling the emissions of CO_2_ from soil and floodwater in intermittently draining rice paddy soil. The soil CO_2_ flux rates ranged from −0.45 to 8.62 µmol.m^−2^.s^−1^ during the rice-growing season. The net effluxes of CO_2_ from the paddy soil were lower when the paddy was flooded than when it was drained. The CO_2_ emissions for the drained conditions showed distinct diurnal variation with a maximum efflux observed in the afternoon. When the paddy was flooded, daytime soil CO_2_ fluxes reversed with a peak negative efflux just after midday. In draining/flooding alternating periods, a sudden pulse-like event of rapidly increasing CO_2_ efflux occured in response to re-flooding after draining. Correlation analysis showed a negative relation between soil CO_2_ flux and temperature under flooded conditions, but a positive relation was found under drained conditions. The results showed that draining and flooding cycles play a vital role in controlling CO_2_ emissions from paddy soils.

## Introduction

Increases in the emission of greenhouse gases such as carbon dioxide (CO_2_), methane (CH_4_), and nitrous oxide (N_2_O) from soil surface to the atmosphere have been a worldwide concern for several decades [1]–[3]. CO_2_ is recognized as a significant contributor to global warming and climatic change, accounting for 60% of global warming or total greenhouse effect [4]. Measuring the soil CO_2_ efflux is crucial for accurately evaluating the effects of soil management practices on global warming and carbon cycling. Temporal variations in soil CO_2_ flux have been observed in almost all ecosystems [5],[6]. Soil CO_2_ fluxes are usually higher during warm seasons and lower during cold seasons [7], [8]. The seasonal variation is driven largely by changes in temperature, moisture, and photosynthate production [5], [9], [10]. The main factors controlling seasonal variations in soil CO_2_ flux may depend on the type of ecosystems and the climate.

The increase in population in areas where rice is the main cultivated crop has led to the increase in worldwide area under rice cultivation by approximately 40% over the last 50 years [11]. In particular, Asian countries (China, India, Indonesia, etc.) have accounted for approximately 90% of the total global area under rice cultivation for the last 50 years [11]. Rice paddies in monsoonal Asia play an important role in the global budget of greenhouse gases such as CH_4_ and CO_2_ [12], [13]. Carbon emisions (esp. CH_4_) from rice paddies are expected to be a long-term contributor to greenhouse gases, perhaps increasingly over the past 5000 years [14]. Efforts have been made recently to model carbon emissions based on the history and archaeology of rice cultivation in Asia. However, since these emissions from rice cultivation vary a great deal, this poses a major challenge in modeling this phenomenon [15]. As a result, experimental research from rice paddies assumes greater importance. Many of the factors controlling gas exchange between rice paddies and the atmosphere are different from those in dryland agriculture and other ecosystems because rice is flooded during most of its cultivation period. The dynamics of soil CO_2_ fluxes in a paddy field differs significantly from that in fields with upland crop cultivation in which aerobic decomposition process is dominant [6], [16], [17]. Field studies designed to measure soil CO_2_ fluxes and improve our understanding of the factors controlling the fluxes are thus needed.

Intermittent draining and flooding, which is one of the most important water management practices in rice production, was found to be the most promising option for CH_4_ mitigation also [18], [19]. Mid-season aeration was also found to be one of the basic techniques for raising rice yields in China [20] and was widely adopted in rice cultivation where irrigation/drainage system was well managed. The management induced change of anaerobic and aerobic conditions results in temporal and spatial (vertical, horizontal) variations in reduction and oxidation (redox) reactions affecting the dynamics of organic and mineral soil constituents [21], [22]. Thus, intermittent drainage with increased impacts can strongly affect soil CO_2_ emissions [6], [16]. However, the mechanism of CO_2_ exchange between rice paddies and the atmosphere is not fully understood. For example, using eddy covariance measurements, Miyata et al. [16] found a significantly larger net CO_2_ flux from the rice paddy soil to atmosphere when the field was drained compared to when it was flooded. These differences in the CO_2_ flux were mainly due to increased CO_2_ emissions from the soil surface under drained conditions resulting from the removal of diffusion barrier caused by the floodwater. The existence of floodwater, anaerobic soil, or changes in the micrometeorological environment with flooding influences root activity, photosynthesis, and respiration of rice plants [23]. Activity of aquatic plants such as algae in the floodwater may also affect CO_2_ exchange between rice paddies and the atmosphere [22]. Most of the data obtained so far were not sufficiently detailed to examine the influence of these factors on the CO_2_ exchange in rice paddies.

The scale and dynamics of growing-season CO_2_ emissions from paddy fields have been documented mostly through flux measurements made with low time resolution using manual chambers [6], [16], [17]. In this study, we report a data set that extends hourly CO_2_ flux measurements during the rice-growing season in 2011 to improve the understanding of the process controlling CO_2_ exchanges in rice paddy soils. The measurements were used to assess the role of floodwater in controlling the exchanges of CO_2_ from the paddy soil. The objectives of this study were to: (i) analyze seasonal and diurnal variation of soil CO_2_ fluxes in rice paddy fields in the Yangtze River valley; and (ii) determine the effects of related environmental factors associated with flooding and draining cycles in paddy soils on CO_2_ flux from the soil surface.

## Materials and Methods

### Site Description

**Figure 1:**
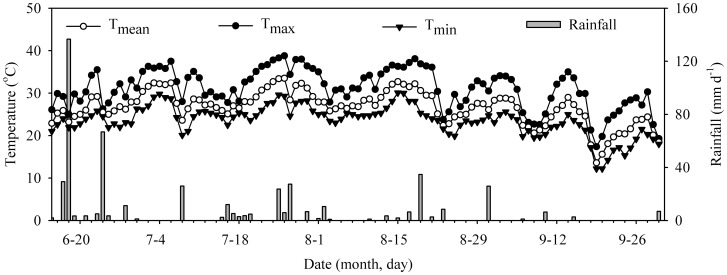
Air temperature records and rainfall events at the study site during the experimental period. (T_mean_: mean temperature; T_max_: maximum temperature; T_min_: minimum temperature).

Field experiments were conducted over one rice growing season, i.e. from June to October 2011, at Nanhu Agricultural Research Station (30°28′N, 114°25′E, altitude 20 m). The research site is owned by Hubei Academy of Agricultural Sciences. The field studies did not involve endangered or protected species and no specific permits were required for the described field studies. The site lies in a typical area of the humid mid-subtropical monsoon climate in the Yangtze River valley of China. The mean annual temperature of the site is 17°C, the cumulative temperature above 10°C is 5,190°C, and the average annual frost-free period is 276 d. The average annual precipitation is 1,300 mm, with most of the rainfall occurring between April and August. The paddy field soil is a Hydromorphic paddy soil, which is a silty clay loam derived from Quaternary yellow sediment. Some physical and chemical properties of the experimental soil (0–20 cm depth) were: pH, 6.3; organic matter, 30.23 g.kg^−1^; total N, 2.05 g.kg^−1^; available P, 5 mg.kg^−1^; available K, 101 mg.kg^−1^; soil bulk density, 1.26 g.cm^−3^. The experimental site has been under rice-wheat cultivation since last 30 years, where rice is planted from June to October each year and wheat is planted from November to May the following year. Daily meteorological information (including rainfall and temperature) during the 2011 rice-growing season is presented in Fig. 1.

### Field Management

In 2011, rice was transplanted to the paddy field on 15 June with a plant to plant spacing of 20 cm and a row spacing of 27 cm. Irrigation started on 13 June and the field was flooded continuously until 17 July. This was followed by five intermittent flooding and draining cycles, with 3–7 days of flooding and 2–8 days of draining. The field was not irrigated and drained about a month before harvesting. The number of flooded days were 55, while the number of drained days were 53 during the 2011 rice-growing seasons. The depth of standing water during flooding periods was, on average, 10 cm. Before transplanting, base fertilizer – consisting of 36 kg N ha^−1^ in the form of urea (N 46%), 45 kg P_2_O_5_ ha^−1^ in the form of calcium superphosphate (P_2_O_5_ 12%), and 90 kg K_2_O ha^−1^ in the form of potassium sulfate (K_2_O 45%) – was broadcast over the soil, which was then turned over by plowing to transfer the fertilizer to the subsurface (i.e., beyond 20 cm soil depth). Additional nitrogen, in the form of urea, was applied at tillering and heading stages of rice growth at rates of 36 and 18 kg N ha^−1^, respectively. Rice grain was harvested from 1 to 3 October, 2011.

### Measurement of Soil CO_2_ Flux

The soil CO_2_ flux was measured using the soil respiration method, where a cylinder static chamber of 22.5 cm diameter and 30 cm height was placed on the soil. The rate of increase in CO_2_ concentration within the chamber was monitored with an ACE (ADC BioScientific Ltd) automated soil CO_2_ flux system. The automated design means that during analysis cycles, the soil can be exposed to ambient conditions before the chamber closes to take measurements. This means the ACE will continue to collect data without any human intervention for as long as permitted by its battery life. This makes the ACE an ideal research instrument for continuous assessment of below-ground respiration and carbon stores in on-going experiments. Static chambers were inserted to a depth of approximately 7 cm, extending 23 cm above the soil surface to allow placing of the chamber. During the flooding period, the water remained in situ. The time span between chamber contact with the soil and the start of measurements (the deadband) was 20 s; this has previously been determined to be sufficient for pressure equilibration. The measurement time was set to 180 s. The ACE has a highly accurate CO_2_ infrared gas analyzer housed directly inside the soil chamber, with no long gas tubing connecting the soil chamber and no separate analyzer. This ensures accurate and robust measurements, and the fastest possible response times to fluxes in gas exchange. During the soil CO_2_ flux measurements, air temperature within the canopy and soil temperature at 2 cm depth were also recorded by the ACE analyzer unit. And the measurements were made at 1-hour intervals during the rice-growing season. During a 24-hour period, the values were averaged to give the mean daily soil CO_2_ flux. Survey sites of three replications were taken from the experiment plot. Survey sites were located in the space between two rows, and the two sites were located 5–7 m apart. Three ACE stations were connected via an ACE Master control unit. Each CO_2_ flux measurement from the experiment plot was thus an average of three individual measurements.

In order to examine the diurnal soil CO_2_ flux pattern in a paddy field, soil CO_2_ flux as well as canopy air temperature, soil temperature and PAR were also measured simultaneously at 1 hour intervals for 24 hours under both flooded (6/28∼6/29 and 8/14∼8/15) and drained (7/20∼7/21 and 9/4∼9/5) conditions. During these 24 hour periods, the sky was clear and with no clouds.

To study the soil CO_2_ emissions in relation to draining and flooding cycle system, two draining/flooding alternation and circulation periods (7/23∼7/28 and 8/29∼9/4) were tested. We continuously monitored soil CO_2_ fluxes along with air temperature within the canopy and soil temperature before, during, and after each flooding and draining cycle in the experiment paddy soil. Clear days continued during the experiment, but temperature conditions were a little different from day to day. Flooding started at 9 am (09∶00 h) and water depth reached 10 cm around midday. The water level was gradually decreased with cessation of irrigation.

## Results

### Seasonal Variations in Soil CO_2_ Fluxes from Paddy Fields

**Figure 2:**
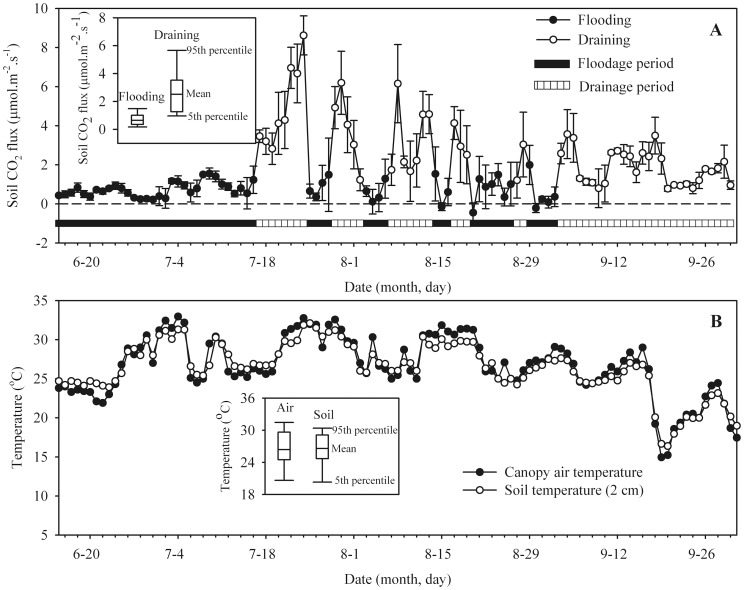
Seasonal variations of soil CO_2_ flux, soil temperature (2 cm) and crop canopy temperature in an intermittent draining and flooding rice paddy. The insets indicate box-plot of soil CO_2_ fluxes under flooded and drained conditions, and box-plot of canopy air and soil temperature during the 2011 rice-growing season.

The daily course of soil CO_2_ flux rate is shown in Fig. 2A, while Fig. 2B shows the air temperature within the canopy and soil temperature (2 cm). The soil CO_2_ flux rates ranged from −0.45 to 8.62 µmol.m^−2^.s^−1^, exhibiting a wide seasonal fluctuation during the rice-growing season. The soil CO_2_ fluxes were generally low at the rice seedling stage, when it remained at about 0∼1µmol.m^−2^.s^−1^ until the first mid-summer drainage. Then the fluxes increased gradually until the tillering stage, with a midway peak near the end of the first mid-summer drainage. From the tillering stage to the physiological maturity stage (i.e, from July to September), the daily average soil CO_2_ flux rates had a magnitude ranging between 0 and 9 µmol.m^−2^.s^−1^, which then settled at around 1∼3 µmol.m^−2^.s^−1^ until the end of the season. The differences in the rates of soil CO_2_ fluxes between drained and flooded conditions are also shown in Fig. 2a. Mean soil CO_2_ fluxes under flooded conditions was 0.72 (with standard deviation of 0.48) µmol.m^−2^.s^−1^ (n = 55), whereas under drained conditions, the corresponding value was 2.79 (with standard deviation of 1.73) µmol.m^−2^.s^−1^ (n = 53). It is likely that floodwater decreased topsoil diffusivity, and may thus have decreased soil CO_2_ effluxes [24]. Reduction of biological activity under anoxic condition may be another reason for low soil CO_2_ fluxes during the flooding period [22].

The air temperature within the canopy and soil temperature (2 cm) exhibited seasonal patterns similar to soil CO_2_ fluxes. The temperature varied from 15 to 33°C during the whole growing period of rice in 2011. From June to September, the temperature ranged from 21 to 33°C, and several peaks occurred. From the mid of September (9/18) to the day before harvesting (about 15 days), the average temperature of 19.7°C for air temperature within the canopy and 19.8°C for soil temperature (0–2 cm) are shown in Fig. 2.

### Diurnal Patterns of Soil CO_2_ Fluxes in Paddy Fields

**Figure 3:**
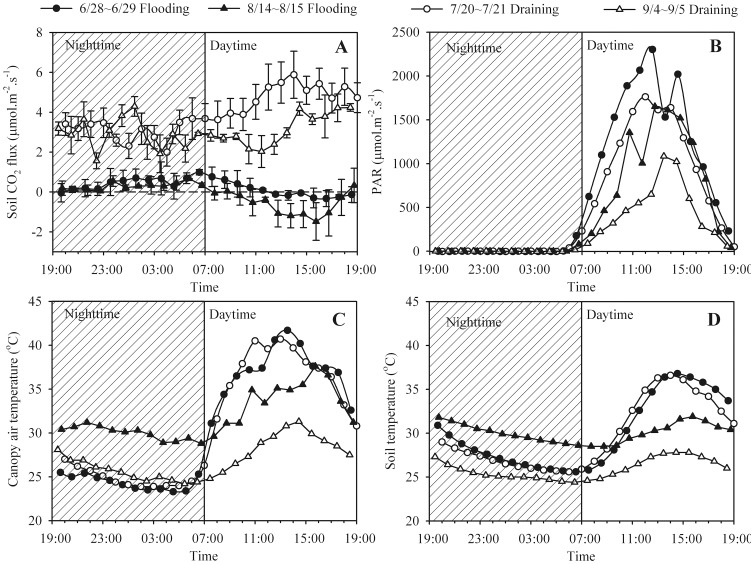
Diurnal patterns of soil CO_2_ flux related to PAR, canopy air temperature and soil temperature (2 cm) under both drained and flooded condtions in a rice paddy.

The diurnal variations in soil CO_2_ fluxes and incident PAR, air temperature within the canopy, and soil temperature under both flooding (6/28∼6/29 and 8/14∼8/15) and draining (7/20∼7/21 and 9/4∼9/5) conditions are shown in Fig. 3. These experiments began in the early evening, running for just under 24 h. Under flooding conditions, fluxes of CO_2_ were, as expected, lower because the diffusivity and biological activity of the topsoil was substantially reduced by floodwater. Initially, there was a slow release of CO_2_ into the atmosphere as a positive efflux settled at around 0–1 µmol.m^−2^.s^−1^ throughout the night. At sunrise the fluxes decreased, even negatively peaked at around 16∶00 (negative values indicate carbon sequestration). This may have been because some aquatic plants, such as algae, inside the floodwater began to photosynthesize again. In contrast, CO_2_ flux under draining conditions was positive and settled around 2∼4 µmol.m^−2^.s^−1^ throughout the night, despite falling temperatures (Fig. 3). After sunrise, CO_2_ fluxes remained positive and increased with temperature, reaching a peak at 2 pm (14∶00 h) before falling again as temperatures declined.

### Soil CO_2_ Fluxes Related to Conversion Processes of Draining and Flooding Cycles

**Figure 4:**
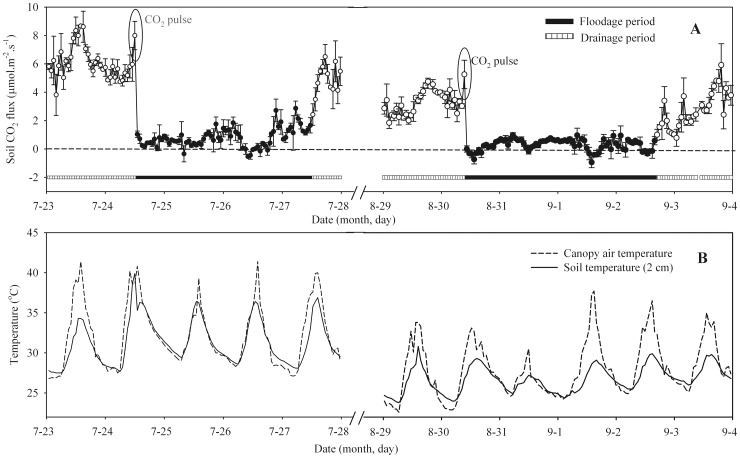
Soil CO_2_ fluxes, soil temperature (2 cm) and canopy temperature before, during, and after the flooding and draining cycle in a rice paddy field.

Fig. 4 shows soil CO_2_ fluxes, canopy air temperature, and soil temperature before, during, and after the flooding and draining cycle. Soil CO_2_ fluxes increased immediately after flooding, and exceeded pre-flooding values by two-thirds. This increase was abrupt and pulselike. Replacement of soil air by water should thus cause an enriched CO_2_ pulse. And then, the soil CO_2_ flux rate subsequently decreased by 70∼90% within only one hour after the water pulse. Within the following days, the CO_2_ fluxes remained at minimum levels (about −2∼2 µmol.m^−2^.s^−1^) during flooding. As standing water declined and eventually disappeared, the CO_2_ fluxes gradually increased and finally reached to maximum levels (about 6∼8 µmol.m^−2^.s^−1^). This indicates that draining and flooding cycles play vital roles in controlling CO_2_ emissions in a paddy soil.

### Variability of Soil CO_2_ Fluxes Related to Temperature

**Figure 5:**
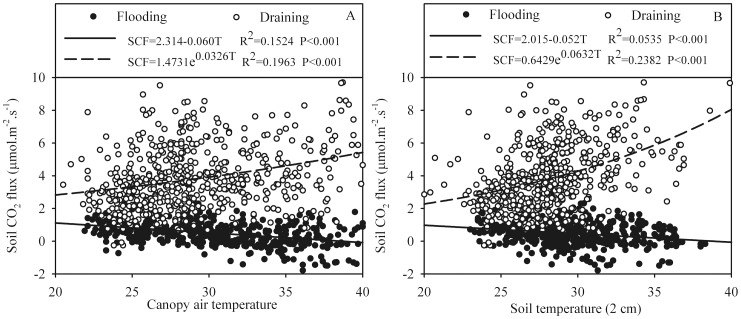
Relationship between soil CO_2_ fluxes and temperature under both flooded and drained conditions. The solid lines represent the regression functions under flooded conditions, and the dashed lines represent the regression functions under drained conditions. (SCF: soil CO_2_ fluxes; T: temperature).

Temperature has a marked effect on CO_2_ emissions from the soil surface. To study the relationship between soil CO_2_ flux rates and temperature, two environmental temperatures (air temperature within the canopy and soil temperature) were tested in this study (Fig. 5). Linear and exponential regression analysis were used to model the influence of temperature on soil CO_2_ flux rates under both flooded and drained conditions. Negative linear correlations between temperature and soil CO_2_ fluxes were found under flooded conditions (R^2^ = 0.1524, P<0.001 and R^2^ = 0.0535, P<0.001 for canopy air and soil temperatures, respectively), presumably because standing water limited soil CO_2_ emissions. On the contrary, soil CO_2_ flux rates increased as an exponential function of temperature under drained conditions (R^2^ = 0.1963, P<0.001 and R^2^ = 0.2382, P<0.001 for canopy air and soil temperatures, respectively).

## Discussion

**Figure 6:**
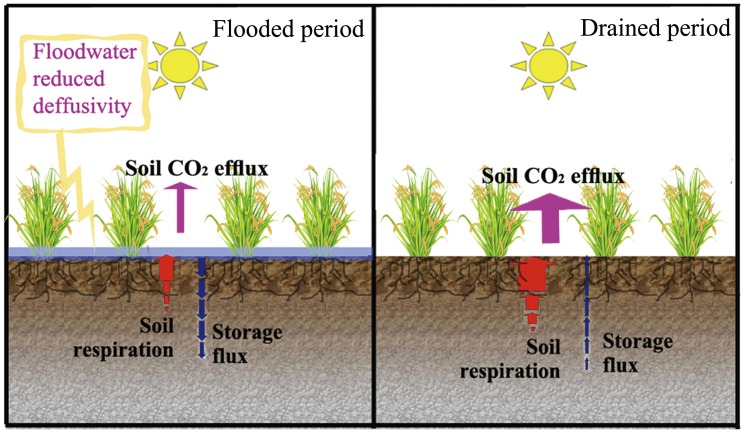
Schematic comparison of soil CO_2_ flux processes under the flooded and the drained conditons in rice paddies.

Previous research had revealed that water management systems show the highest potential in controlling CH_4_ emissions [25]. CH_4_ emissions were higher under continuous flooding than intermittent draining practices [26], [27], while they declined during the drainage period to near zero and increased after re-flooding [28]. Drainage during the rice cultivation period significantly increased CO_2_ emissions in our study, while CH_4_ emissions were clearly reduced and has been shown by other research [18], [29]. Miyata et al. [16] also found that flooded or drainage conditions of paddy soils had strong effects not only on CH_4_ emissions but also on CO_2_ emissions. Lower CH_4_ emissions due to water drainage may increase CO_2_ emission. However, during the submerged period of paddy rice cultivation, CO_2_ production in the soil is severely restricted under flooding condition. This effect can be explained with two basic mechanisms [8], which could be observed in a paddy soil (Fig. 6). First, flooding a field for subsequent rice cultivation cuts off the oxygen supply from the atmosphere and the microbial activities switch from aerobic (i.e. oxic condition) to facultative (i.e. hypoxic condition) and to anaerobic (i.e. anoxic condition) conditions [22]. As a consequence, biological activity reduction under anoxic condition, rather than completely, inhibits CO_2_ production. At the same time, water replaces the gaseous phase in the soil pores. Since CO_2_ diffusion rates in water are four orders of magnitude lower than those in air, a part of the produced CO_2_ is stored in the soil. Hence, the soil CO_2_ fluxes can be dramatically reduced by flooding during the paddy rice cultivation [6], [16], [23]. Results from the present study provide indirect support for this conclusion, since the soil CO_2_ flux rates under flooded conditions were significantly lower than those observed under drained conditions (Fig. 2).

Our study also demonstrated that, in rice fields exposed to intermittent flooding and draining cycles, environmental factors regulating diurnal fluctuations in CO_2_ flux are quite different from those governing seasonal variations. Under drainage conditions, soil CO_2_ flux showed a single peak at 2 pm (14∶00 h), and was lowest in the wee hours. This is in agreement with patterns recorded in forests [5], grassland [30] and dryland areas [31]. Furthermore, correlation analysis revealed that canopy air temperature and soil temperature explained most of the diurnal fluctuations in soil CO_2_ flux. In contrast, soil CO_2_ flux during the flooding period fluctuated within ±2 µmol.m^−2^.s^−1^ and soil CO_2_ flux rates had small negative values in the daytime (i.e., the paddy soil was obviously a net CO_2_ sink.), although soil CO_2_ fluxes were positive throughout the night. This occurred primarily because of the layer of standing water, which is the habitat of bacteria, phytoplankton, macrophytes and small fauna. The photosynthesis process of these aquatic organisms affects ecosystem respiration [22].

Sudden pulse-like events of rapidly increasing CO_2_ efflux occur in soils under paddy fields in response to re-flooding after draining. Similarly, an abrupt rise in near-surface soil moisture due to precipitation can cause an instantaneous soil respiration pulse [24], [32]. Soil respiration is shown to respond rapidly and instantaneously to the onset of rain and return to the pre-rain rate shortly after the rain stops [32]. The likely reason for this is that CO_2_ is heavier than air and accumulates by gravitation within the air spaces of the soil. Replacement of this gaseous carbon by dilution will not occur without water and, unstirred by turbulent mixing, accumulation of CO_2_ within the soil will increase. A sudden flooding might simply seal the soil pores, replace the captured CO_2_ by water, and release it back into the air [33]. These occurrences, termed “Birch effect”, can have a marked influence on the ecosystem carbon balance [34], [35]. Indeed, this transient effect was observed in several studies at the ecosystem [36] and soil [37] scales. **On the other hand**, our analysis indicates that soil CO_2_ flux was gradually increased during flooding to draining conversion processes. Response of soil CO_2_ flux rates to these processes can be viewed in terms of increased diffusivity due to decrease in water filled pore space. Besides this general effect of soil aeration on soil CO_2_ flux, the higher soil respiration rates during the drainage periods may have resulted from the higher physiological activity of microorganismsin not limiting soil oxic conditions [22].

We examined possible seasonal effects of temperature on soil CO_2_ flux and found significant relation between the two under both flooded and drained conditions, but with widely differing mechanisms. In the present study, we found a negative relation between temperature and soil CO_2_ flux, as long as soil CO_2_ diffusivity is limiting as is the case during flooding period. An alternative explanation is based on the photosynthetic activity of the aquatic botany. The periods with the high photosynthetic active radiation are associated with conditions of high temperature in daytime (Fig. 3). Under drainage conditions, when soil aeration is assumed to be almost constant, soil temperature is considered to be a major control of soil CO_2_ flux. Also the positive exponential relationship between soil CO_2_ flux and temperature were observed during drainage period (Fig. 3). The results under drained conditions are similar to those of previous studies of CO_2_ flux. For example, Chang et al. [38] found strong relationships between CO_2_ flux and soil temperature and indicated that the rates of CO_2_ emission increased exponentially with increases in soil temperature. Liu et al. [7], on the other hand, reported a significantly (P<0.01) linear relationship between soil CO_2_ flux and soil temperature at a depth of 5 cm.

### Conclusions

From the comparison of soil CO_2_ fluxes under draining and flooding conditions we conclude that: (1) the net effluxes of CO_2_ from the paddy soil were lower when the paddy was flooding than when it was draining, (2) the enhanced fluxes of CO_2_ from the draining soil were due to removal of the barrier to gas transport from the soil surface to the air caused by the floodwater, and (3) there was a negative relation between soil CO_2_ flux and temperature under flooding condition, whereas a positive relation under draining condition. The present study also showed how flooding and draining cycles affect the exchanges of CO_2_ during the rice cultivation period. We need more measurements for multiple years to assess the long-term effect of an intermittent flooding and draining practice on the exchanges of CO_2_ in rice paddy fields.
